# Clinical Significance of Carnitine in the Treatment of Cancer: From Traffic to the Regulation

**DOI:** 10.1155/2023/9328344

**Published:** 2023-08-10

**Authors:** Raheleh Farahzadi, Mohammad Saeid Hejazi, Ommoleila Molavi, Elahe Pishgahzadeh, Soheila Montazersaheb, Sevda Jafari

**Affiliations:** ^1^Hematology and Oncology Research Center, Tabriz University of Medical Sciences, Tabriz, Iran; ^2^Molecular Medicine Research Center, Tabriz University of Medical Sciences, Tabriz, Iran; ^3^Department of Pharmaceutical Biotechnology, Faculty of Pharmacy, Tabriz University of Medical Sciences, Tabriz, Iran; ^4^Department of Pharmaceutical Biotechnology, School of Pharmacy, Shahid Beheshti University of Medical Sciences, Tehran, Iran; ^5^Nutrition Research Center, Tabriz University of Medical Sciences, Tabriz, Iran

## Abstract

Metabolic reprogramming is a common hallmark of cancer cells. Cancer cells exhibit metabolic flexibility to maintain high proliferation and survival rates. In other words, adaptation of cellular demand is essential for tumorigenesis, since a diverse supply of nutrients is required to accommodate tumor growth and progression. Diversity of carbon substrates fueling cancer cells indicate metabolic heterogeneity, even in tumors sharing the same clinical diagnosis. In addition to the alteration of glucose and amino acid metabolism in cancer cells, there is evidence that cancer cells can alter lipid metabolism. Some tumors rely on fatty acid oxidation (FAO) as the primary energy source; hence, cancer cells overexpress the enzymes involved in FAO. Carnitine is an essential cofactor in the lipid metabolic pathways. It is crucial in facilitating the transport of long-chain fatty acids into the mitochondria for *β*-oxidation. This role and others played by carnitine, especially its antioxidant function in cellular processes, emphasize the fine regulation of carnitine traffic within tissues and subcellular compartments. The biological activity of carnitine is orchestrated by specific membrane transporters that mediate the transfer of carnitine and its derivatives across the cell membrane. The concerted function of carnitine transporters creates a collaborative network that is relevant to metabolic reprogramming in cancer cells. Here, the molecular mechanisms relevant to the role and expression of carnitine transporters are discussed, providing insights into cancer treatment.

## 1. Introduction

Carnitine is an amino acid-derived compound found in almost all cells in the body. Carnitine facilitates the transfer of acyl groups across the cell membranes for *β*-oxidation and ATP production. Carnitine has direct/indirect modulatory effects on several physiological systems, such as the neural system [1]. Although brain cells have low levels of *β*-oxidation, carnitine is actively transported across the blood–brain barrier and accumulates in neural cells [1]. Approximately 75% of carnitine in the human body is derived from dietary sources, such as animal products such as red meat and dairy products (with high amine content), and 25% is synthesized endogenously from lysine and methionine in the liver and kidneys.

Carnitine homeostasis is achieved by a balance between endogenous synthesis, intestinal absorption, and renal reabsorption, indicating that carnitine homeostasis does not simply rely on maintaining a constant level. Various tissues require different amounts of carnitine for survival. For instance, in the testis, carnitine is at the highest level for sperm maturation. Skeletal muscle and myocardium are also carnitine-dependent tissues because fatty acid oxidation (FAO) is the primary energy source for meeting the energy demand [2].

Due to the impermeability of the mitochondrial inner membrane to fatty acyl-CoA thioesters, the specialized transporting system has evolved to transport fatty acids across mitochondrial membranes. Components of this system include carnitine palmitoyltransferase 1 (CPT1) and 2 (CPT2), the carnitine–acylcarnitine carrier (CAC), and the carnitine acetyltransferase (CrAT, also known as CAT). The latter allows the export of the FAO-produced acetyl-CoA as acetylcarnitine from mitochondria to the cytoplasm. Carnitine and acyl groups can be converted to acylcarnitine by carnitine CPTI (also known as CPTA1) in the cytoplasm. In the mitochondrial matrix, CPT2 catalyzes the conversion of acylcarnitines to carnitine and acyl-CoAs. Acyl-CoAs undergo *β*-oxidation to generate acetyl-CoA that enters the tricarboxylic acid cycle (TCA). In the heart, acylcarnitine may provide an immediate energy source by FAO and the release of carnitine and transfer of the acyl group to CoA for subsequent *β*-oxidation in the TCA. As a result, maintaining carnitine homeostasis is crucial for cellular metabolism due to its shuttling role in FAO [3].

Over the past decade, reactive oxygen species (ROS) and free radicals have gained more considerable attention owing to their harmful pathological effects. ROS can trigger oxidative stress and damage various cellular components such as DNA, proteins, and lipids [4]. The body continuously produces ROS during normal physiological processes, which are neutralized by various antioxidant defense mechanisms [5]. Carnitine plays a crucial role in protecting cells from free radicals and the harmful effects of ROS and retards the progression of chronic diseases and aging [6–8]. In addition, carnitine protects the mitochondrial membrane integrity against ROS attack and reduces lipid peroxidation [9]. Carnitine exerts its protective effect against oxidative damage by regulating the function of enzymes involved in the defense system, including superoxide dismutase (SOD), glutathione peroxidase (GPx), and catalase (CAT) [10]. In addition, Nicassio et al. [11] showed that carnitine has the potential to restore age-related alterations in mitochondrial dynamics and function in aged animal models. In this regard, carnitine can normalize age-associated alterations and disorders primarily caused by free radicals.

**Figure 1:**
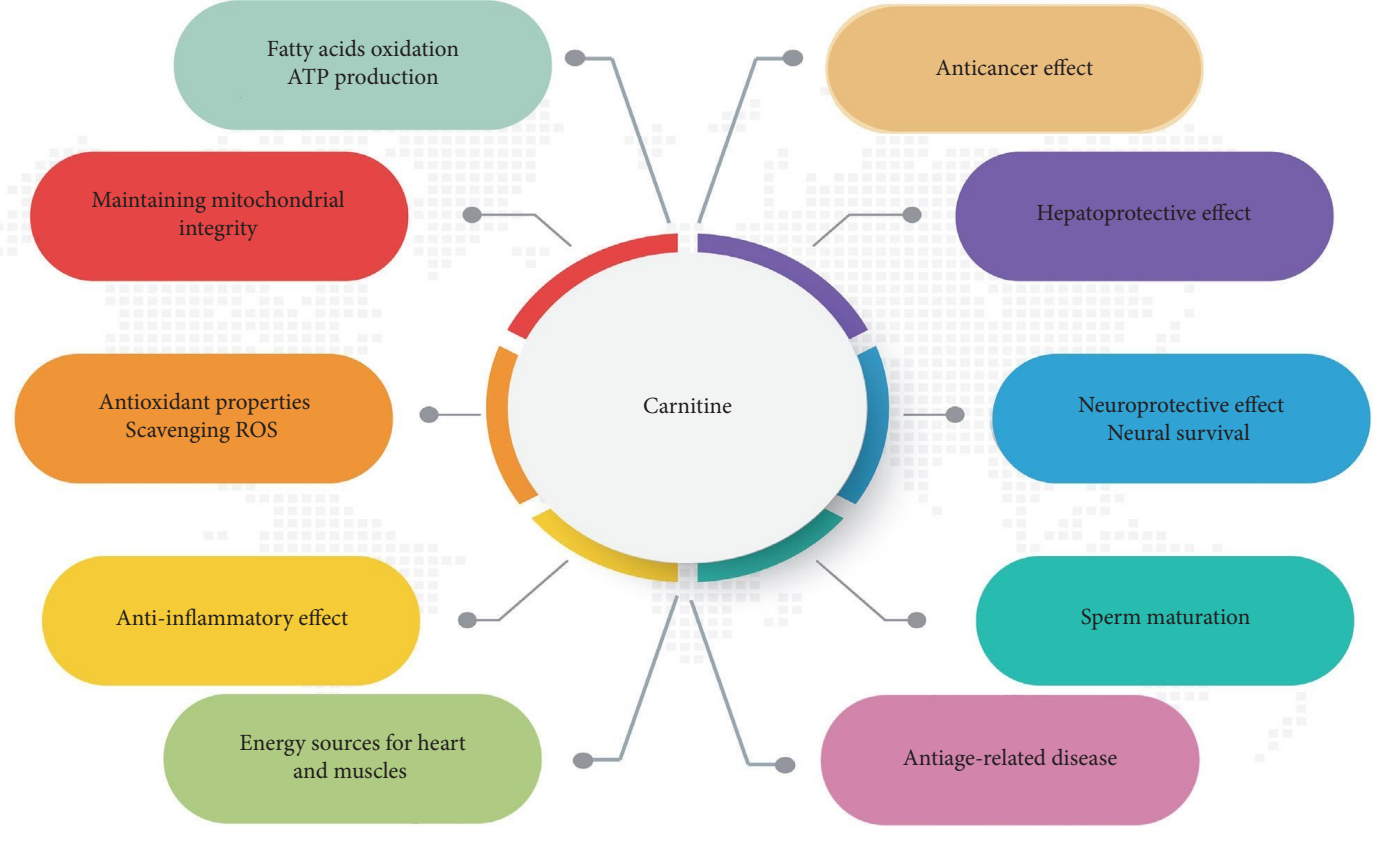
The carnitine's role in various cellular processes.

Several studies have reported the beneficial therapeutic effects of acetylcarnitine in various neurological disorders such as Alzheimer's disease (AD), Parkinson's disease, epilepsy, and depression in the elderly [12]. Magi et al. [13] showed that carnitine could ameliorate neuronal damage in glyceraldehyde-induced AD phenotype. Glyceraldehyde is used as a glycolysis inhibitor. According to these results, carnitine improves cell survival in the neurodegenerative context of AD. In addition, carnitine can improve intracellular ATP levels and mitochondrial function and reduce the generation of ROS in mitochondria [13]. In an animal model of liver injury, carnitine blocked the essential pathways involved in nitric oxide synthase activity by inhibiting the nuclear factor-kappa B (NF-*κ*B) and phosphatidylinositol 3-kinase/Akt pathways. These findings indicate the hepatoprotective effects of carnitine [14]. Carnitine has several pleiotropic roles in health and disease (Figure 1). Considering these notions, it is not surprising that carnitine traffic can be altered under other pathological conditions such as cancer.

Carnitine plays a significant role in fatty acid shuttling into the mitochondria for FAO and ATP generation. All cells reprogram their metabolic demands under harsh conditions to support proliferation, growth, and survival [15]. This metabolic plasticity is more prominent in cancer cells than in normal cells. In other words, cancer cells rewire their metabolism to meet the energy requirements and biosynthetic intermediates for survival and replication. Metabolic plasticity can maintain the integrity of cancer cells under hostile and hypoxic conditions [16]. In addition to the glycolytic pathway, cancer cells mediate diverse metabolic strategies such as FAO to fulfill their requirements. In this context, carnitine plays a pivotal role in the metabolic plasticity of cancer cells by interacting with fundamental pathways, mediators, and regulators to supply energy and biosynthetic molecules [17]. With these considerations, a proper understanding of the interconnection between tissues, cellular subcompartments, and membrane transporters in carnitine traffic is needed to realize the possible role of carnitine in cancer. Given the importance of cancer as the leading cause of premature mortality worldwide, we focused on the relationship between alterations in carnitine traffic and metabolic plasticity in cancer.

## 2. Carnitine Sources and Pharmacokinetics

As mentioned above, carnitine in human is synthesized endogenously in addition to being taken up exogenously [18]. Carnitine biosynthesis from lysine and methionine is carried out in the liver and kidney through a multistep process. Lysine provides the carbon chain of carnitine, and methionine provides methyl groups. During the biosynthesis of carnitine, lysine residues undergo *N*-methylation using *S*-adenosylmethionine (as a methyl donor) to form 6-*N*-trimethyl-lysine (TML) residues, as the first metabolite in the biosynthesis of carnitine. TML is converted to carnitine in four enzymatic reactions. The first step is TML hydroxylation, which is mediated by TML dioxygenase, and generates 3-hydroxy-6-*N*-trimethyllysine (HTML). This reaction requires *α*-ketoglutarate as a necessary cofactor. *α*-ketoglutarate is converted into succinate and CO_2_ is released. The second step is aldolytic cleavage, which is mediated by the HTML aldolase to yield 4-*N*-trimethylaminobutyraldehyde (TMABA) and glycine. The third reaction involves the dehydrogenation by TMABA dehydrogenase (TMABADH) to form 4-*N*-trimethylaminobutyrate (butyrobetaine). In the final reaction, butyrobetaine is hydroxylated at carbon 3 to from carnitine.

On the other hand, carnitine absorption is mediated by both active transport and passive diffusion. The bioavailability of carnitine varies between 54% and 87% and depends on the ingestion quantity. Carnitine concentrations in skeletal muscle are 100 times greater than those in plasma, indicating an active transport system for this compound [19]. Intestinal absorption of carnitine reaches saturation level following administration of 2 g of carnitine, and the concentration reaches to its maximum level at 3.5 hr and then gradually decreases [20]. Approximately 80% of myocardial carnitine is absorbed through circulation [21], whereas in other tissues, carnitine is taken up by the carnitine/organic cation transporter (OCTN) family [22]. Most of the carnitine in the body is excreted in urine as carnitine or acylcarnitine; however, very little is lost in bile. The plasma and tissue concentrations of carnitine are highly conserved because of tubular reabsorption by 98% of the filtered free carnitine. The majority of acylcarnitine is excreted in the urine, allowing for the excretion of abnormal metabolites. Therefore, the plasma level of carnitine is 25–50 *μ*M in the healthy subjects, whereas the plasma level of acetylcarnitine is in the range of 3–6 *μ*M [23].

## 3. Carnitine Traffic and Transporters in Cancers

**Figure 2:**
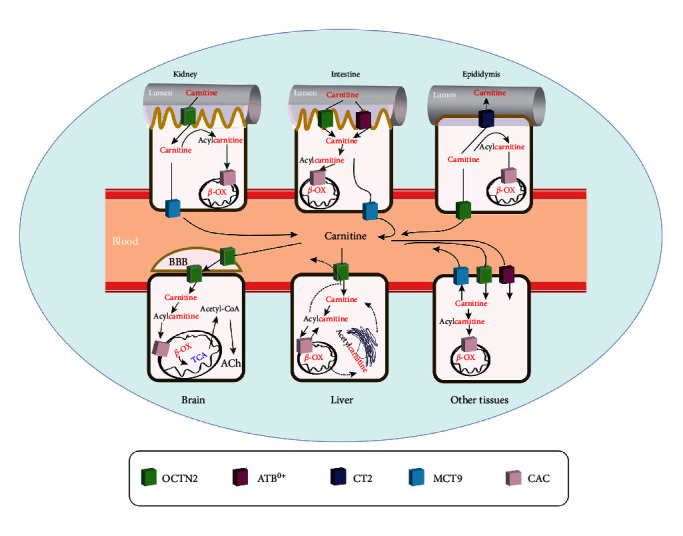
Involvement of various transporters in carnitine traffic. Straight arrows depict carnitine traffic, and dotted arrows notify the transport of some other substrates involved in carnitine traffic. In this scenario, OCTN2, ATB^0,+^, and CAC mediate carnitine transport for *β*-oxidation. OCTN2, organic cation transporter novel 2; ATB^0,+^, amino acid transporter B^0,+^; CAC, mitochondrial carnitine/acylcarnitine carrier; CT2, carnitine transporter 2; MCT9, monocarboxylate transporter 9; *β*-ox, mitochondrial *β*-oxidation.

The carnitine transporter network is tightly regulated via a dedicated system within the tissues and subcellular compartments of the human body. Indeed, carnitine trafficking is mediated by various membrane transport proteins. Multiple membrane transporters contribute to the transfer of carnitine and its derivatives across cellular membranes (Figure 2). The solute carrier (SLC), the plasma membrane transporters OCTN1 (with low affinity), OCTN2/SLC22A5, carnitine transporter 2 (CT2)/SLC22A16, monocarboxylate transporter 9 (MCT9)/SLC16A9, and ATB^0,+^/SLC6A14 mediate the flux of carnitine. Besides, CAC/SLC25A20 (mitochondrial carnitine/acylcarnitine carrier), an essential component of the carnitine shuttle, catalyzes the exchange of free carnitine with acylcarnitine in mitochondria [24]. OCTN3/SLC22A21 is a carnitine-specific transporter in mice but not in humans [25]. In the following section, the action of carnitine transporters is explained, and their relevant role in cancer is discussed (Table 1).

### 3.1. OCTN1/SLC22A4

OCTN1 is a transporter belonging to the SLC22 family in humans. Noteworthy, OCTN1 and OCTN2 are expressed in human, while OCTN3 is expressed only in mice and rats. OCTN1 is highly expressed in the kidneys but at a lower level in the bone marrow, spinal cord, skeletal muscle, heart, the trachea, lung, liver, pancreas, spleen, intestine, uterus, and neural cells [42]. OCTN1 is ubiquitously expressed with polyspecific substrate recognition potential. Accordingly, information regarding the role of OCTN1 in carnitine trafficking is controversial, even if its physiological significance remains unclear. Carnitine and acetylcarnitine can be transported by OCTN1 with low affinity because carnitine is not the main substrate. OCTN1 can transport acetylcholine, a physiological substrate of the nonneuronal cholinergic system, under physiological and pathological conditions [43]. Accumulating evidence shows that mutated OCTN1 is associated with susceptibility to Crohn's disease [42, 44].

OCTN1 is expressed in various cancer cell lines. OCTN1 contributes to sporadic colorectal cancer (CRC) in the early stage [45], and its genetic variants may predict malignant progression in patients with inflammatory bowel disease [46]. The contributory role of OCTN1 in cancer metabolism does not rely on poor transportation of carnitine. Indeed, OCTN1 exerts its effect by facilitating the uptake of several anticancer agents, such as camptothecin, cytarabine, daunorubicin, and mitoxantrone [26, 47]. OCTN1 is thought to mediate the uptake of the mushroom-derived compound ergothioneine, which may act as an antioxidant in tissues exposed to ROS [27].

### 3.2. OCTN2/SLC22A5

OCTN2 is another membrane transporter that belongs to the SLC22 family contributing to carnitine trafficking in various tissues [48]. The function of this transporter is clearly defined in experimental models using the HEK293 cell line with constant overexpression of human OCTN2 (OCTN2-HEK293) and proteoliposomes harboring human OCTN2 [49]. Carnitine absorption from the intestinal tract and reabsorption from the kidney are mainly controlled by the plasma membrane OCTN2, which shows the highest affinity for carnitine among other transporters. OCTN2 also plays a substantial role in carnitine trafficking between the tissues. In addition to the kidney and intestine, OCTN2 is expressed in various tissues, such as the heart, liver, skeletal muscle, brain, testis, mammary gland, and placenta. OCTN2 plays an important role in transferring carnitine from the maternal blood across the placenta into fetus. The transporter has a role in the secretion of carnitine into milk through the mammary gland [50].

Noteworthy, OCTN2 is expressed in areas where carnitine is physiologically essential [51, 52]. A high level of carnitine is found in the testes and epididymis, reaching up to 60 mM in the epididymal lumen. The massive gradient of carnitine concentration between the epididymal lumen and plasma is attributed to the presence of OCTN2 on the blood side [53] and CT2 on the lumen side of epithelia [25] (Figure 2).

OCTN2 transports carnitine in a sodium-dependent manner and accumulates carnitine inside cells, resulting in a concentration gradient between the blood and the intracellular space. Given the prominent role of OCTN2 in carnitine absorption and distribution, its deficiency leads to a rare inherited metabolic disorder in newborns called primary carnitine deficiency (PCD) [54]. This disease is characterized by a decrease in intracellular carnitine levels, accumulation of acyl-CoA esters, and inhibition of mitochondrial acyl transport. Carnitine plays a prominent role in transferring long-chain fatty acids across the inner membrane of mitochondrial membrane for fatty acid oxidation. Reduced carnitine transport leads to a broad spectrum of symptoms, including acute metabolic derangement, cardiac symptoms, myopathic manifestations, skeletal muscle weakness, hypoglycemia, and hyperammonemia. However, it has been revealed that high doses of carnitine can improve the clinical manifestations of these disorders [55, 56]. Meanwhile, other carnitine transporters with lower affinities may partially relieve the symptoms described above. In this regard, the ATB^0,+^, MCT9, and OCTN1 may compensate for the lack of OCTN2 [57].

Carnitine and its acyl derivatives are the main substrates of OCTN2, and their transport is mediated by Na^+^ cotransport. OCTN2 can also facilitate the transport of organic cations such as tetraethylammonium (TEA) in a Na^+^-independent manner. Based on these data, it can be assumed that the active sites for organic cations and carnitine overlap but are not identical [58]. A variety of transport modes have been defined for OCTN2. OCTN2 can catalyze Na^+^-dependent symport and/or antiport modes. This transporter facilitates the efflux of carnitine derivatives favored by their outward concentration gradients. Consequently, the actual transport path (e.g., symport or antiport) of OCTN2 may rely on its isoforms and tissue expression. Accordingly, the possible transport pathways and tissue distribution explain the role of OCTN2 in carnitine traffic within the intestine, distribution to body districts, and kidney reabsorption/excretion [43]. In line with FAO, OCTN2 is also controlled by transcription factors that regulate lipid-metabolizing events. One example is peroxisome proliferator-activated receptor (PPAR) *α*, which directly regulates OCTN2 gene expression and FAO [59]. In addition, PPAR*γ* can regulate the expression of OCTN2 by binding to the PPAR-responsive segment within the first intron [60]. OCTN2 plays a critical role in fatty acid metabolism, as evidenced by human pathologies resulting from altered transporter functions [28, 61]. In some cases, the symptoms of the inherited disease can be mimicked by the administration of drugs that interact with OCTN2 as an off-target [62]. Notably, a chronic inflammatory status can contribute to the emergence of pathological conditions such as cancer, highlighting the importance of OCTN2 and carnitine as the main substrates. Therefore, there is a need to elucidate the interplay between the expression and function of OCTN2 with lipid metabolism in cancer. Several lines of evidence indicate that the altered expression of this transporter is linked to cancer development and progression. Intriguingly, overexpression or downregulation of OCTN2 relies on the tumor's carbon source to generate energy [29, 30, 63]. Cancers growing in a lipid-rich niche display enhanced lipid utilization, providing survival advantages for cancer cells [64, 65]. A recent review showed that OCTN2 is overexpressed in various types of cancers, including ovarian, endometrial, renal, and pancreatic cancers, as well as glioblastoma multiforme (GBM). OCTN2 is highly expressed in glioblastoma, even when neurons do not normally use fatty acids to obtain energy. It can be assumed that in glioblastoma with aggressive features, an increase in cellular carnitine content is necessary to fulfill the high energy demand for cell growth and proliferation. FAO can serve as an alternative energy source to enhance glioblastoma progression and proliferation [57]. A high level of OCTN2 expression in GBM patients is associated with a poor outcome because silencing OCTN2 by siRNA-mediated activity could decrease tumor cell viability [30].

Studies have shown that estrogen signaling coordinates the overexpression of OCTN2 in metastatic breast cancer patients with estrogen receptor-positive (ER^+^) cancers. This was confirmed by silencing the estrogen receptor in ER^+^ breast cancer cells, which decreased the expression of OCTN2. Indeed, the knockdown of SLC22A5 by siRNA-mediated inhibition could reduce carnitine intake. It led to the accumulation of lipid droplets and suppression of breast cancer cells [66, 67]. Based on these findings, it can be inferred that OCTN2 could be a therapeutic target for ER^+^ breast cancer. In contrast, CRC exhibits low expression level of OCTN2 [28]. Likewise, Scalise et al. [68] reported that human papillomavirus (HPV)-mediated carcinoma showed reduced expression of this transporter. In good agreement, the naturally harboring HPV16 cell line exhibited downregulation of OCTN2, which was attributed to methylation of the promoter region. In cancer, epigenetic modulation of OCTN2 may be applied to enhance the effectiveness of anticancer compounds [68, 69]. Given the regulatory role of PPAR*γ* in OCTN2 expression, luteolin, a natural agonist of PPAR*γ*, could increase OCTN2 expression in a time- and dose-dependent manner. Accordingly, luteolin-mediated activity could potentiate the sensitivity of CRC cells to chemotherapeutic agents such as oxaliplatin, as OCTN2 is a determinant factor in oxaliplatin uptake [60]. Kou et al. [70] utilized carnitine-conjugated nanoparticles to improve the efficacy of paclitaxel delivery in glioma cells. Furthermore, imatinib, which is used as first-line treatment in patients with chronic myeloid leukemia (CML), is transported into cells via OCTN2 and OCTN1 [31]. As a result, a link has been found between rs2631365-TC within the promoter region of OCTN2 and failure of imatinib treatment [71], highlighting the development of personalized therapy.

### 3.3. CT2/SLC22A16

Similar to OCTN2, the plasma membrane CT2 belongs to the SLC22 family of transporters. The expression of CT2 is restricted to healthy tissues but is highly expressed in several cancers. Intriguingly, the cancer-associated expression of CT2 is observed in tissues that generally do not express this transporter. This indicates the metabolic rewiring of cancer cells concerning carnitine-related metabolism. Overexpression of CT2 has been detected in acute myeloid leukemia (AML), as this cancer type depends on FAO. Considering the dysregulation of CT2 in cancer, Wu et al. [32] reported that knocking down CT2 expression leads to reduced growth and viability in cancer cells. It has been shown that CT2 was significantly upregulated in gastric cancer compared to healthy stomach tissues. An analysis of over 300 patients over 10 years revealed that upregulation of CT2 was associated with poor survival among patients with stomach cancer. In this respect, CT2 can be regarded as a drug target for gastric cancer [33]. Besides, CT2 can be exploited for drug delivery. Consistent this notion, Okabe et al. [34] reported that CT2 mediates doxorubicin uptake in cancer cells. According to their results, CT2 overexpressing cells underwent apoptosis after exposure to doxorubicin (2 *μ*M), whereas control cells did not. As a result, CT2 may be a new candidate for regulating the doxorubicin influx and increasing intracellular doxorubicin accumulation.

Beyond this, several reports have revealed the capacity of this transporter to transport anticancer agents with high affinity. Using whole-exome sequencing analysis, Novak et al. [72] reported that CT2 was lost in 54% of the patients with diffuse large B-cell lymphoma. Indeed, lack of remission or early relapse is a crucial clinical issue in these patients. This can be attributed to the loss of CT2 transporter in the cell membrane, which impairs drug uptake into cancer cells. In another similar report, Sagwal et al. [73] provided evidence regarding the knockdown of CT2 and the subsequent inhibition of cytotoxic effects in malignant melanoma. In other words, they found that increased intracellular doxorubicin in melanoma cells was mediated by the upregulation of the CT2 transporter. In addition, it has been found that cisplatin is a substrate of CT2. Another study reported that elevated levels of CT2 expression in lung cancer are associated with higher accumulation of cisplatin (CDDP) in cancer cells. In contrast, CT2 downregulation results in resistance to CDDP by decreasing intracellular platinum concentration [74]. The functional characterization of CT2 is still in its very early stages; however, its unusual tissue distribution indicates that CT2 maintains the epididymal gradient of carnitine to serve as an osmolyte and FAO cofactor [33, 75].

### 3.4. ATB^0^,^+^/SLC6A14

ATB^0,+^/SLC6A14, a sodium and chloride symporter, is a unique plasma membrane transporter with broad substrate specificity. ATB^0,+^ mediates the transport of neurotransmitters, osmolytes, and all amino acids except for acidic ones (e.g., aspartate and glutamate) [76]. ATB^0,+^ is expressed in the lung and intestinal epithelia. It is intriguing to note that ATB^0,+^ recognizes carnitine as a substrate with a lower affinity than the OCTN2 transporter. Interestingly, ATB^0,+^ can be expressed in tissues, such as epithelial cells of the normal human airway [77], eye [78], and mammary glands [79], allowing carnitine distribution within these tissues. ATB^0,+^ transports carnitine in a Na^+^- and Cl^−^-coupled form [80]. Unlike the OCTN2 transporter that mediates the transport of carnitine derivatives, ATB^0,+^ is able to transport only carnitine and propionyl carnitine [81]. Similar to other plasma membrane transporters, the overexpression of ATB^0,+^ has been detected in several types of human cancers, thereby becoming a hallmark of cancer. This could be due to the high capacity of ATB^0,+^ to transport amino acids. In light of this, ATB^0,+^ can be considered as druggable target [36, 82]. Many reports have shown that ATB^0,+^ is a delivery system for many drugs and prodrugs [83, 84]. Kou et al. [37] used carnitine-conjugated nanoparticles targeting OCTN2 and ATB^0,+^ to deliver chemotherapeutic agents to colon cancer. Colocalization experiments using fluorescently-labeled nanoparticles confirmed the contribution of OCTN2 and ATB^0,+^. Indeed, colon cancer cells exhibited upregulated levels of OCTN2 and ATB^0,+^ compared with normal colon cells. In this context, carnitine-conjugated nanoparticles can be used to selectively deliver chemotherapeutic agents. Based on their results, carnitine-conjugated nanoparticles were taken up by OCTN and ATB^0,+^ in a cell-specific manner [37]. In another similar study, amino acid-based triptolide prodrugs delivered anticancer compounds in pancreatic cancer to target ATB^0,+^ [38]. Indeed, this transporter is highly expressed in pancreatic cancer cells; as a result, high ATB^0,+^-mediated uptake results in cancer cell death.

### 3.5. Carnitine/Acylcarnitine Carrier (CAC)

CAC/SLC25A20 is a mitochondrial transporter belonging to the SLC25 family. CAC exerts its effect in a ping-pong-mediated manner in the carnitine/acylcarnitine exchange. In other words, CAC transports acylcarnitines into the mitochondria in exchange for free carnitines. CAC plays a crucial role in the regulation of acyl unit influx into the mitochondrial matrix for FAO [85]. Several studies have confirmed that posttranslational modifications in CAC regulate its transport activity. In this context, lysine acetylation of CAC prevents carnitine entry into the mitochondria, thus having a detrimental impact on FAO [86]. Few reports have examined the interplay between CAC alterations and cancer development. As mentioned earlier, the use of fatty acids in prostate cancer cells is higher than in normal cells. Valentino et al. [39] provided evidence that miRNAs are involved in the deregulation of mitochondrial FAO by modulating the carnitine system. They found the aberrant expression of miR-129-5p, miR-124-3p, and miR-378 in human prostate cancer specimens compared to normal prostate specimens. The deregulation of these miRNAs increases the expression and function of CPT1 and CAC in prostate cancer cells. These findings suggest that forced expression of these miRNAs can reduce the expression levels of CPT1A, CAC, and CAT, negatively affecting FAO. In addition, the mitochondrial carnitine system is a potential druggable target for the treatment of prostate cancer. Moreover, significant deregulation was detected in carnitine–acylcarnitine in urine samples from patients with bladder cancer compared to healthy controls [40].

### 3.6. MCT9/SLC16A9

In the case of MCT9 transporter, there is a lack of information. MCT9 is a ubiquitous transporter with the highest expression levels in the kidneys and adrenal glands. Therefore, its role in regulating carnitine traffic is plausible [87]. On the other hand, the presence of MCT9 in the basolateral membrane of enterocytes may provide carnitine transport into the bloodstream. However, no definitive data exist regarding the involvement of MCT9 in carnitine distribution under physiological conditions. In addition, the human umbilical vein endothelium expresses MCT9, which may play a role in carnitine-induced proinflammatory responses. Consistent with this notion, MCT9 expression is enhanced by tumor necrosis factor-*α* (TNF-*α*). This leads to carnitine accumulation within endothelial cells to stimulate FAO and energy production, which is necessary for maintaining inflammatory responses [88].

## 4. Carnitine and Cancer

Patients with cancer were found to be susceptible to carnitine deficiency. The caloric intake of cancer patients is often impaired, while their metabolic demands are increased. Aside from that, pharmacological therapy in cancer patients can interfere with carnitine synthesis, absorption, and excretion [89, 90]. Carnitine deficiency has been reported in chronic illnesses such as cancer [91]. Decreased serum levels of carnitine have been detected in multiple cancers, including endometrial cancer, breast cancer, CML, and pediatric cancer [92–95]. Numerous studies have reported beneficial effects of carnitine in patients with advanced cancer. In a literature review by Radkhouy et al. [96], the beneficial anticancer effect of carnitine was revealed in colon cancer, as evidenced by the prevention of tumor growth. Baci et al. showed that the administration of acetylcarnitine had an angiopreventive effect on prostate cancer cells. Aberrant expression of cytokines/chemokines in prostate cancer can govern progression, invasion, and angiogenesis [97]. High expression of chemokine receptor 4 (CXCR4), an angiogenic factor, is associated with metastatic behavior and poor survival. Acetylcarnitine exerts its anticancer effect by acting on the cytokine/chemokine axis of prostate cancer [98, 99].

Cachexia is a multifactorial syndrome characterized by loss of skeletal muscle mass with or without loss of fat mass. This condition cannot be fully compensated by conventional nutritional support, resulting in progressive functional defects in these patients. In a study done by Mitchell et al. [100], it was shown that pancreatic cancer patients exhibit cachexia at the time of diagnosis. Patients with cancer cachexia are resistant to dietary interventions; however, carnitine supplementation could improve the quality of life and body mass. Impairment of FAO can be attributed to the reduced activity of CPTI and CPTII in the liver. CPTI and CPTII play a vital role in the development of cancer cachexia. Accumulating evidence has revealed the importance of carnitine molecules in fatty acid metabolism. In cancer cachectic mice, Liu et al. [101] found a decreased levels of serum-free carnitine and acetylcarnitine with downregulated mRNA levels of CPTI and CPTII. In addition, a hepatic reduction in CPTI activity was detected. According to their results, oral administration of carnitine at a dose of 18 mg/kg significantly restored CPT activity and downregulated the serum levels of interleukin-6 (IL-6) and TNF-*α* in animal models. With this respect, it can be assumed that carnitine-mediated amelioration is associated with CPT regulation in the liver [101]. In a cachectic mouse model of colon cancer, Jiang et al. [102] showed that oral administration of carnitine at a dose of 9 mg/kg/day ameliorated the cachexia parameters. Carnitine can also decrease the elevated serum levels of IL-6 and TNF-*α* in cancer cachectic mice [102]. Data from a similar recent study have indicated the potential benefits of carnitine in cancer therapy. Their findings revealed that carnitine improved cancer cachexia in an animal model through the Akt/FOXO3/MaFbx and p70S6K pathways. Carnitine also decreased IL-1 and IL-6 serum levels, which are responsible for the progression of cancer-associated cachexia [63]. In addition, it has been shown that carnitine administration can alleviate disorders of lipid metabolism. Beyond this, carnitine can decrease the serum levels of hepatic enzymes, including aspartate aminotransferase (AST), alanine aminotransferase (ALT), and triglyceride (TG), which are significantly elevated during irregular feeding in cancer patients [103].

Metabolic reprogramming and increased ATP demand are well-established hallmarks of cancers [104]. FAO plays a crucial role in providing ATP, NADH, FADH_2_, and NADPH, thus providing survival benefits to cancer cells. CPTI is a rate-limiting FAO enzyme that contributes to cancer metabolic adaptation, and its overexpression can fuel tumor growth in numerous tumor types [105]. CPTI can crosstalk with various cellular signaling pathways involved in cancer pathogenesis. In this regard, inhibition of CPTI may suppress cancer development [106]. From these studies, it can be inferred that carnitine has a beneficial impact on the management of cancer symptoms.

**Figure 3:**
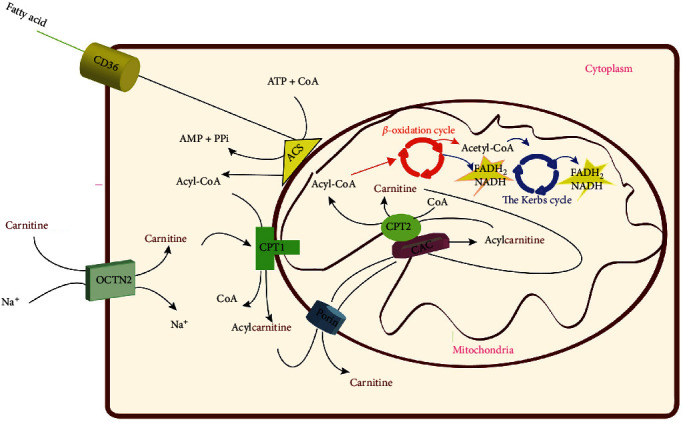
An overview of the carnitine system in the mitochondria. OCTN2 mediates the carnitine uptake into cells. FAO substrates are crossed from the external environment by CD36. In the cytosol, acetyl-CoA synthetase (ACS) catalyzes the conversion of fatty acids into acyl-CoA and then are converted into the carnitine derivatives by carnitine palmitoyltransferase 1 (CPT1), located within the outer mitochondrial membrane. Acylcarnitine is translocated into the mitochondrial matrix by the cooperation of CPT1, carnitine palmitoyltransferase 2 (CPT2), and carnitine/acylcarnitine carrier (CAC). In the matrix, CPT2 catalyzes the conversion of acylcarnitines to acyl-CoAs and undergoes *β*-oxidation to generate acetyl-CoA. Finally, acyl-CoA enters to TCA cycle to produce ATP.

As discussed earlier, cancer cells require more energy than normal cells. In other words, energy demand increases with tumor aggressiveness and malignancy [107]. Normal cells meet their energy requirements through TCA and oxidative phosphorylation in the mitochondria (Figure 3) [108]. Under aerobic conditions, normal cells meet their energy demands through glycolysis in the cytosol, followed by oxidative phosphorylation within the mitochondria. Cancer cells alter their metabolism to support growth, survival, proliferation, and long-term maintenance [109]. Indeed, cancer cells prefer to obtain energy from glycolysis even in the abundance of oxygen, a phenomenon referred to as the “Warburg effect.” Glycolysis is much faster (100 times) than oxidative phosphorylation, even though the energy production is much lower. These events occur in the cytosol, even in the presence of functional mitochondria and abundant oxygen. Cancer cells bypass the mitochondrial respiratory chain, which synthesizes ATP. Such metabolic reprogramming has been observed in various cancer types. [110–112]. In addition to aerobic glycolysis, cancer cells can also stimulate fatty acid biosynthesis and glutamine consumption. Glutamine is considered the second crucial growth-supporting substrate in cancer cells. During metabolic adaptation, most cancer cells utilize glucose and glutamine as their primary carbon sources [113]. In cancer cells, mitochondrial function is not entirely impaired, and oxidative phosphorylation and TCA are still functioning [114]. In addition, there is increasing evidence that some cancers exhibit dual capacities for glycolysis and oxygen-consuming metabolism. Notably, metabolic flexibility exists in diverse cancers and cancers of the same type but at various stages. Metabolic plasticity can promote cancer cells growth, invasion, and metastatic behavior [16].

In the case of lipid metabolism, there is convincing evidence that some cancers increase fatty acid utilization, whereas others decrease it. Considering the role played by carnitine in FAO, proper intervention is needed to control carnitine levels and/or its trafficking. It is well known that any alteration in FAO may impact the availability/dynamic of structural membrane lipids, lipid synthesis, and energy production [115, 116]. Consistent with this notion, alteration of lipid metabolism has been reported in CRC [117, 118]. Cancer cells undergo metabolic reprogramming to proliferate abnormally and inhibit cell death signaling. In addition, cancer cells rely on FAO to enhance their growth, evasion, invasion, drug resistance, and metastasis. FAO is the primary energy source for cancer cells; thereby, high levels of FAO enzymes exist in cancer cells [119, 120]. FAO substrates are obtained from the external environment through specific transporters such as CD36, which contributes to fatty acid uptake and storage in adipose tissues. CD36-mediated lipid uptake is required for sufficient ATP synthesis in mitochondria through FAO [121, 122] (Figure 3).

Lipid metabolic reprogramming is considered an essential feature of cancer cells, and fatty acid metabolism plays a critical role in lipid metabolism. De novo lipogenesis in fatty acid synthesis relies mainly on acetyl-coenzyme A (acetyl-CoA). In contrast, exogenous fatty acid intake requires transport molecules such as CD36 and fatty acid-binding proteins (FABPs) [123, 124]. CD36 is a transmembrane molecule that plays an essential role in fatty acid uptake and mitochondrial FAO [125].

Lipolysis produces enormous amounts of fatty acids, which are necessary for the survival of cancer cells. Generally, de novo lipogenesis and exogenous fatty acids are increasingly required to supply energy for oncogenic signals involved in cancer progression and tumorigenesis [126]. As a result, CD36 and FABP are overexpressed in cancer cells. CD36 differs from other transporters that regulate carnitine and acylcarnitine traffic in that; it deals with hydrophobic fatty acid molecules rather than participating directly in the carnitine network [127]. Lipid droplets are dynamic cytoplasmic organelles that contribute to cell signaling, membrane trafficking, lipid metabolism, and inflammatory marker production. These molecules can modulate crosstalk between tumor cells and other cell types in the tumor niche. Lipid droplets are another source of lipids for FAO [128].

Aside from the direct advantage of producing ATP from fatty acids, FAO also manages oxidative stress induced by electron transport. Acetyl-CoA is the end product of FAO, which enters the TCA cycle and forms an isocitrate molecule. Isocitrate is then oxidized by cytosolic isocitrate dehydrogenase 1 to generate *α*-ketoglutarate and NADPH, which are required for ROS detoxification. Next, *α*-ketoglutarate enters back into the TCA cycle. The requirement of NADPH is evidenced by the presence of various pathways in cancer cells that are activated to meet their demands. NADPH can also be derived from malic enzyme activity and the pentose phosphate pathway. Notably, these pathways are highly upregulated by the oncogene AKT, which acts upstream of the transcription factor Nrf2. Moreover, AKT mediates activation of the nicotinamide adenine dinucleotide kinase (NADK) to phosphorylate NADH-generating NADPH [129, 130]. According to evidence, lipid metabolism in cancer is not restricted to the oxidative route; cancer cells committed in anabolic metabolism to generate new building blocks that maintain tumor cell growth and proliferation. Since lipids are essential building blocks for cell membranes, they are listed as vital molecules required by proliferating cells. Therefore, cancer cells not only use FAO to oxidize fatty acids and energy production in the mitochondria, but they also utilize acetyl-CoA for endogenous synthesis of fatty acids in the cytosol, using NADPH as an essential anabolic cofactor. Indeed, this is a futile cycle of concomitant fatty acid synthesis and degradation in cancer cells, which is forbidden by “canonical” biochemical reactions [131, 132]. This condition is caused by the removal of CPT1 inhibition through the specific downregulation of acetyl-CoA carboxylase, with a strong decrease in the level of malonyl-CoA (an allosteric inhibitor of CPT1). In other words, malonyl-CoA is a potent regulator of CPT1, the enzyme that transfers the acyl group into the mitochondria where it is oxidized. Acetyl-CoA carboxylase produces malonyl-CoA, which regulates the activity of CPT1. Downregulation of acetyl-CoA carboxylase decreases the level of malonyl-CoA and releases the inhibition of mitochondrial fatty acid *β*-oxidation, enhancing the oxidation of fatty acids [131]. In metabolic networks that promote FAO, carnitine traffic can be regarded as the startup process. In this context, FAO cannot occur without appropriate carnitine traffic.

### 4.1. Carnitine and Oxidative Stress

Patients with advanced cancer experience oxidative stress, as evidenced by elevated ROS and decreased GPx levels [133]. Oxidative stress and anorexia/cachexia syndrome alone or in combination exhibit predictive clinical outcomes in patients with cancer. Cachexia is a multifactorial syndrome, and targeting only oxidative stress is inadequate and likely to achieve limited therapeutic efficacy. Therefore, antioxidants should be administered as essential agents of a multitargeted combined therapy in cancer cachexia, which can be the most successful strategy in this syndrome [134]. ROS and nitrogen species are synthesized at low levels in skeletal muscle cells and are required for normal force production. In contrast, higher levels of these agents may inhibit the tissue antioxidant capacity, resulting in oxidative stress [135]. Oxidative imbalance contributes to cancer-induced cachexia and significantly affects proteins as major targets in tissues [136]. Higher levels of ROS within myofibers trigger pathophysiological signaling and induce apoptosis and proteolysis [137, 138]. There is also evidence that cachexia is related to a reduction in muscle glutathione (GSH) levels. Relying on this, tumor-bearing mice with weight loss showed lower GSH levels and higher GSH disulfide/GSH ratios [139]. Treatment with carnitine restored the reduced levels of muscular glutamate and GSH and enhanced plasma glutamate levels in tumor-bearing rodents [140, 141].

The antioxidant activity of carnitine is well established in humans and rats. In this regard, Elkomy et al. [142] reported protective effects of carnitine against cisplatin-induced hepatotoxicity and nephrotoxicity in rats. Oxidative stress is the central mechanism underlying in this toxicity. Oral administration of carnitine (100 mg/kg body weight) in rats could ameliorate cisplatin-induced oxidative stress, as evidenced by restoring the activities of oxidative/antioxidant parameters. Carnitine was found to be effective in cisplatin-induced cardiotoxixity in terms of mitigating malondialdehyde (MDA) levels and elevating the levels of GSH and CAT in hepatic and renal tissues compared to those treated with cisplatin alone [142]. In addition to these side effects, cardiotoxicity has been reported in patients receiving cisplatin. In this context, acetylcarnitine may have a cardioprotective effect against cisplatin-induced cardiotoxicity at the oxidative stress level via modulation of SOD levels [143]. Collectively, carnitine was found to be able to neutralize oxidative and nitrosative stress mediated by various mechanisms, including direct scavenging of free radicals (e.g., superoxide and hydrogen peroxide), inhibition of ROS-generating enzymes such as xanthine oxidase and NADPH oxidase, and more importantly, by upregulation of antioxidant enzymes such as CAT, SOD, glutathione-S-transferase (GST), and GPx [10].

## 5. Carnitine and Inflammation

As mentioned above, cancer cachexia is a wasting syndrome that occurs in patients with advanced cancer. This disorder leads to an increase in the levels of proinflammatory cytokines such as TNF-*α*. It has been reported that TNF-*α* may have a pivotal role in regulating body mass and muscle catabolism [144]. Based on the data from a systematic review and meta-analysis of three databases, anti-TNF increased body weight and body mass index (BMI). Accordingly, blocking TNF-*α*-mediated weight loss could be a potential pharmacological option for treating cancer cachexia [145]. Carnitine has been shown to mitigate inflammatory responses under pathological conditions. Recent evidence has shown the potential benefits of carnitine in the treatment and prevention of cancer cachexia. However, the precise underlying mechanism remains unknown. In a mouse model of cachexia, carnitine reduced the serum levels of IL-1 and IL-6, which are possible inducers of cancer cachexia, with slight effects on TNF-*α* [63].

In a meta-analysis, carnitine reduced serum inflammatory cytokines, including IL-6, TNF-*α*, C-reactive protein (CRP), and MDA, with elevated SOD levels in healthy individuals or patients with specific disorders [146]. Another study reported that the anti-inflammatory effects of carnitine are mediated by a decrease in circulating proinflammatory cytokines and the regulation of CPT activity. CPT plays a pivotal role during the regulation of carnitine in the liver inflammatory response. Carnitine ameliorated the liver inflammatory response via CPT I-dependent PPAR*γ*-NF-*κ*B signaling [147]. Carnitine also mediates its anti-inflammatory activity via the downregulation of NF-*κ*B, which decreases the expression of proinflammatory cytokines [148].

Given the capacity of carnitine to reduce inflammation in animal models, it can be hypothesized that carnitine is a novel therapeutic intervention for alleviating inflammatory reactions in COVID-19 infection [149]. Massive cytokine release occurs during the severe form of COVID-19. It is evident from the various studies; carnitine has a modifying role in improving pathogenic processes. In fact, carnitine is an immune system regulator by modulating proinflammatory cytokines such as TNF-*α*, IL-1, and IL-6. In addition, carnitine has shown a protective effect against COVID-19-induced cardiotoxicity, mainly caused by inflammatory cytokines [150, 151]. Carnitine supplementation is effective in hemodialysis children by alleviating inflammatory markers such as IL-6 [152, 153]. In a study by Emran et al. [154], the cardioprotective effect of carnitine was demonstrated in a rat model of myocardial infarction. Based on their findings, carnitine remarkably decreases the infiltration of inflammatory-related cells and restores heart tissue architecture. This is due to a reduction in the inflammatory response via downregulation of TNF-*α* and IL-1*β* expression to nearly normal levels [155]. Moreover, oxidative status is improved by decreasing ROS and increasing endogenous antioxidant levels [154]. The role of carnitine in reducing inflammatory markers, in part, can be attributed to its ability in decreasing ROS production. Considering the superior effects of carnitine, it can be assumed that it is an ideal option for alleviating cancer-induced inflammation and oxidative stress.

## 6. Conclusion

This review addresses the metabolic plasticity of cancer cells and the roles of carnitine during this process. Carnitine plays a pivotal role in mammalian physiology; hence, fine regulation is needed to maintain carnitine homeostasis, especially in tissues with high FAO rates. Carnitine is an essential metabolite that facilitates shuttling of fatty acids into mitochondria for *β*-oxidation. Cancer cells rely heavily on FAO to maintain growth and malignancy. In addition, carnitine contributes to the regulation of the acyl-CoA/CoA balance, which influences lipid and carbohydrate metabolism. The data reported here show that membrane transporters are unequivocally involved in carnitine traffic. The scenario is discussed in terms of the deregulation of carnitine traffic/homeostasis in human cancers. In this regard, transporters can be potential anticancer therapeutic targets, together with sugar and amino acid transporters, already used as drug targets. The expression levels of OCTN2 and CT2 are altered in human cancers; therefore, carnitine supply is strictly controlled during cancer development. Indeed, CT2 has minimal tissue distribution. In contrast, it is widely expressed in cancers, even in tissues which CT2 is not ordinarily present. Thus, blocking FAO is a potential therapeutic strategy for impairing energy production in cancer cells. There is also evidence that CT2 and OCTN2 are involved in the uptake of various anticancer agents; hence, altering CT2 and OCTN2 expression may explain the efficacy of treatments. On the other hand, increased oxidative stress and inflammatory status are related to cancer symptoms; therefore, therapeutic strategies to alleviate such harmful effects may be helpful in cancer patients. Considering carnitine's ability to improve oxidative/inflammatory profiles in cancer patients, more clinical trials are required to delineate the optimal administration of carnitine as a therapeutic compound. Therefore, extensive research concerning molecular mechanisms responsible for carnitine hemostasis, traffic, expression, and function are opening future perspectives for cancer therapy. The present data indicate that carnitine is an appealing complementary intervention for cancer patients. In addition, due to the deregulation of carnitine transporters, they can be used as potential anticancer targets. Notably, blocking FAO can impair energy production in cancer cells, resulting in beneficial impacts on cancer patients.

## Figures and Tables

**Table 1:** Important carnitine transporters, substrates, and related functions in cancers.

Carnitine transporter	Substrate	Cancer type	Mechanism of action	References
OCTN1/SLC22A4	Carnitine and acylcarnitine with low affinity, acetylcholine, ergothioneine, TEA	Acute myeloid leukemia (AML)	Transport of anticancer drugs such as daunorubicin and mitoxantrone	[26, 27]
OCTN2/SLC22A5	Carnitine, acetylcarnitine with the highest affinity, TEA, *γ*-butyrobetaine	High-grade serous epithelial ovarian cancer, breast cancer, lung adenocarcinoma, glioma, endometrial cancer, renal cancer, pancreatic cancer	Maintenance of carnitine homeostasis/*β*-oxidation, carcinogenesis and chemoresistance development, transport several anticancer drugs, such as imatinib	[28–31]
CT2/SLC22A16	Carnitine	Human epithelial ovarian cancer, gastric cancer, AML	Upregulation in gastric cancer, involving in drug delivery such as doxorubicin	[32–35]
ATB^0,+^/SLC6A14	Carnitine with low affinity, all amino acids except aspartate and glutamate	Pancreatic cancer, colon cancer, estrogen receptor-positive breast cancer	Upregulated in solid tumors, used for drug delivery	[36–38]
CAC/SLC25A20	Carnitine, acylcarnitine	Bladder cancer prostate	Deregulated in tumor tissues of bladder cancer, overexpression in prostate cancer	[39, 40]
MCT9/SLC16A9	Carnitine	Breast cancer	Reduced survival	[41]
